# Structural Study of the Photo-Mediated Growth of Silver Nanoprisms

**DOI:** 10.3390/molecules25225413

**Published:** 2020-11-19

**Authors:** Matti Knaapila, Ulla Vainio, Sophie E. Canton, Gunnel Karlsson

**Affiliations:** 1 Department of Physics, Technical University of Denmark, 2800 Kgs. Lyngby, Denmark; 2 Hitachi Hich-Tech Analytical Science Finland Oy, 02631 Espoo, Finland; 3 ELI-ALPS, ELI-HU Non-Profit Ltd., 6720 Szeged, Hungary; sophie.canton@eli-alps.hu; 4 Chemical Center, Lund University, 22100 Lund, Sweden; gunnel.karlsson@chem.lu.se

**Keywords:** nanoprism dispersion

## Abstract

We present a small-angle X-ray scattering (SAXS) study of the anisotropic photoinduced growth of silver (Ag) nanoprisms in aqueous dispersions. The growth of nearly spherical (<10 nm) Ag particles into large (>40 nm) and thin (<10 nm) triangular nanoprisms induced by 550 nm laser is followed in terms of particle size using indirect and direct methods for irradiation times up to 150 min. During the process, the surface-to-volume ratio of the particles decreased. The SAXS data of the initial solution fit well to the model of polydisperse spheres with pronounced average diameters around 7.4 nm and 10 nm. The data after 45 min irradiation fit well to the model containing approximately the same amount of the initial particles and the end product, the nanoprisms.

## 1. Introduction

Metal-based nanoparticles allow structures from well-defined objects to dendrimers [1]. Metal nanoparticles can be grown directly from solutions by several procedures including Turkevich synthesis [2], seed-mediated growth [3] and laser ablation [4,5] among others. Silver and gold nanoprisms are anisotropic nanoparticles that are prepared from colloidal dispersions using a photo-mediated (also known as plasmon-mediated) synthesis [6,7]. These particles have a characteristic triangular morphology, which can be controlled by varying the wavelength of the irradiation [8] or the pH of the reaction solution [9,10]. A wide range of morphologies is accessible, such as decahedras [11] or bipyramids or penta-twinned rods [12,13]. Other syntheses and further modifications are also possible, including galvanic replacement synthesis [14] or the incorporation of nanoprisms into liquid–liquid interfaces and composites [15].

The structure of nanoprisms is usually monitored in their near-native state by cryogenic transmission electron microscopy (or cryo-TEM) which allows direct imaging. However, each image captures only a part of the sample so that obtaining comprehensive statistical information is time-consuming. Furthermore, cryo-TEM requires somewhat complicated sample preparation which may also affect the sample. Small-angle X-ray scattering (SAXS) provides information about the entire dispersion and does not require any sample preparation, thus complementing cryo-TEM. SAXS has already been employed to follow citrate synthesis of gold nanoparticles providing information on precursor and final particle sizes and size distributions [16] as well as on the growth of gold nanoprisms [17]. However, the use of SAXS to study photo-mediated silver nanoprisms is not well developed.

In this paper, we monitor the photo-mediated synthesis of silver nanoprisms using SAXS. Using a selection of models, the formation process from spherical particles to well-defined triangles is followed in terms of particle shape, size and polydispersity. These data combine the single-particle viewpoint and the macroscopic sample viewpoint on nanoprism growth. This gives a clear evidence for the benefits of SAXS studies in nanoprism development.

## 2. Results and Discussion

The structural evolution of the particle growth was studied first by cryo-TEM. Scattering studies were employed to complement electron microscopy and mirror the overall ensemble.

**Figure 1:**
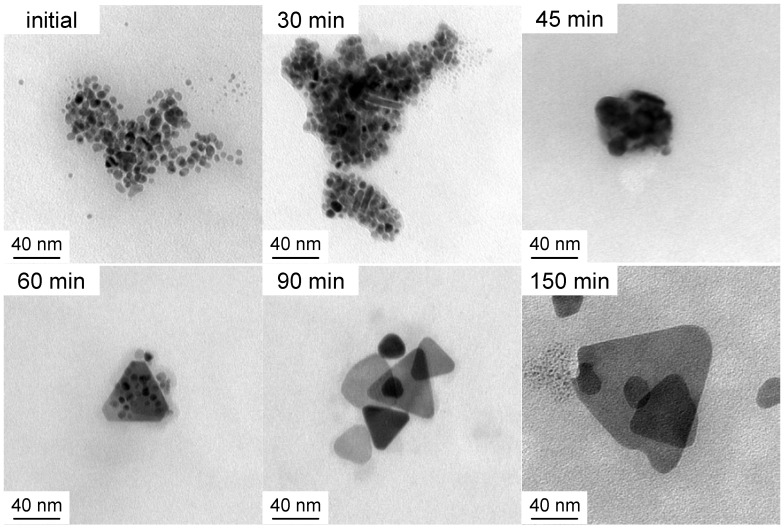
Examples of cryo-TEM images as a function of irradiation time. From left to right: Initial 0 min, 30 min, and 45 min (upper row) and 60 min, 90 min and 150 min irradiated solutions (lower row).

**Figure 2:**
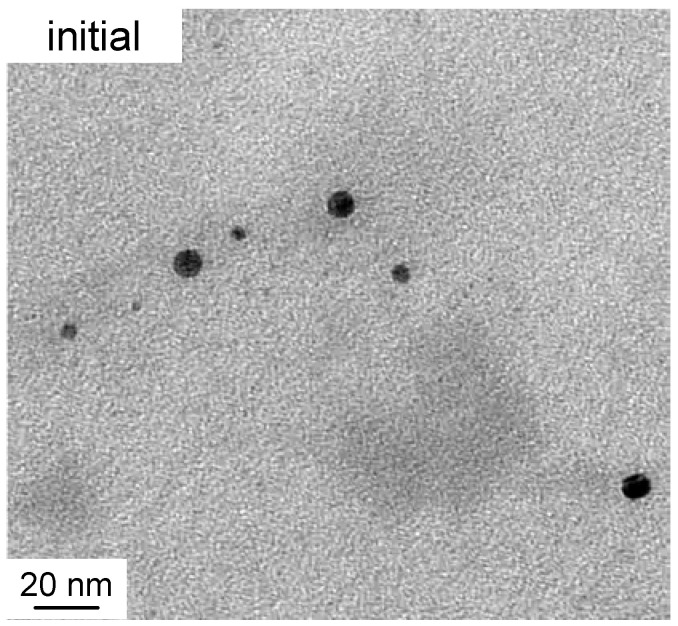
A detail of cryo-TEM image of initial sample showing particles of size 3–8 nm.

Figure 1 shows typical cryo-TEM images of selected solutions with increasing irradiation time. Figure 2 zooms on a detail of the initial sample. All studied samples were polydisperse so no quantitative conclusions may be drawn based on the individual images. However, the following qualitative observations can be made. Initially, as expected, the material consists of nearly spherical nanoparticles (diameter of around 10 nm), which exist both completely separately and in loose aggregates where the individual particles are not fully joined together. Both the individual particles and their loose aggregates were observed up to 75 min irradiation period. After 25–30 min, the particles begin to join together within the previously observed loose groups forming rodlike chains whose length extends up to 40 nm. At the same time, triangle-shaped platelets, also known as nanoprisms, begin to form and their size varies between 20 nm and 120 nm. The size of the prisms increases with the irradiation time so that the larger particles are not observed until 150 min. Even though 40 nm platelets dominate the distribution, all the samples are polydisperse. Therefore, for the present case, it is not possible to distinguish clear bimodal growth and “type I and II” particles as reported by Jin et al. [8]. The representative initial, 45 min and 150 min solutions were subsequently studied with SAXS. The questions are: Is the scattering from all particles indistinguishable or will we rather observe scattering from particle aggregates? If the scattering is indistinguishable, are we able to find out the particle structure and polydispersity distribution?

**Figure 3:**
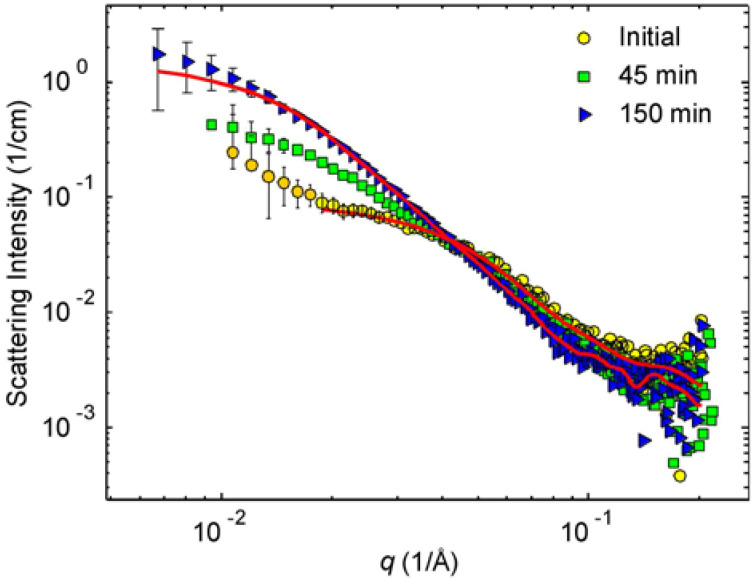
SAXS patterns from initial (yellow circles), 45 min (green squares) and 150 min solutions (blue triangles). Solid lines are the corresponding GNOM fits (see Section 3 for details).

Figure 3 shows the measured SAXS intensities for the initial sample as well as for the 45 min and 150 min irradiated samples corresponding to their colors “yellow”, “green” and “blue”. The attention is placed first on the data of the initial and final, 150 min irradiated sample.

Phenomenologically, the scattering curve of the initial sample consists of two parts, the low q upturn as well as a plateau and a downturn at the higher *q*. We propose that the former part is a tail of the scattering associated with the loose aggregates of initial particles and the latter with separated particles. This idea corresponds to the cryo-TEM result. The literature describes initial particles as spheres [8] and this idea cannot be challenged by the cryo-TEM result. The SAXS data contain a power-law decay that can originate from polydisperse spheres, but also from other particle shapes. The exponent of the power-law cannot be determined reliably because of a strong background and moderate statistics of the data.

The scattering of the 150 min data originates from the nanoprisms. However, as the observation window in our scattering experiment is limited, reaching up to 70 nm, we may not probe the largest prisms with 120 nm edge lengths.

**Figure 4:**
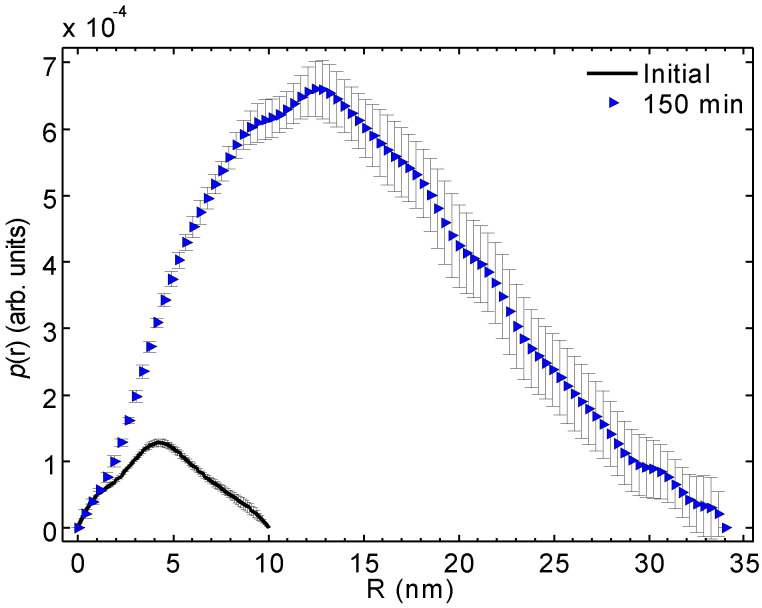
The p(r) functions for the initial (solid line) and 150 min solutions (blue triangles) calculated using the GNOM program.

**Figure 5:**
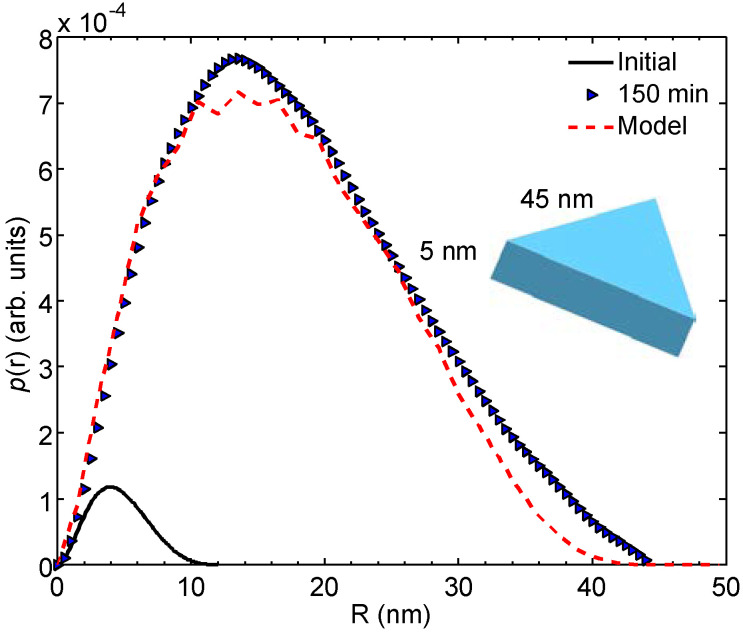
The p(r) functions for the initial (solid line) and 150 min solutions (blue triangles) calculated using the Fourier transform method. The p(r) function calculated for a triangular model prism (red dashed line) is shown for comparison. The model is shown in the inset.

Figure 4 shows the distance distribution functions, p(r) functions, of the initial and 150 min irradiated samples corresponding to the fits calculated by GNOM. Figure 5 plots the corresponding p(r) functions calculated by direct Fourier transform of the same SAXS curves. The calculated distance distribution of a triangular particle model is shown for comparison. Essential structural parameters are listed in Table 1.

The shapes of the p(r) functions determined by these alternative methods are not identical but reasonably similar. Attention should be placed on the shape of the p(r) function of the 150 min irradiated blue solution. The maximum is found on the smaller R side, which indicates that either the sample is somewhat polydisperse (and the smaller part is more populated) or that the particle shape deviates from a sphere. The volume of the triangular particle model (Figure 5) agrees with that determined from SAXS data for the average particle (Table 1). This result is fully consistent with the electron micrographs pointing to a system that is both polydisperse and comprised of flat particles (Figure 1).

Table 1 lists the radius of gyration over all the axes of rotation and maximum diameter of the “average particle”. The particle size calculated from the SAXS data corresponds to the phenomenological majority entities as observed by cryo-TEM. Thus the SAXS data agree with the above-mentioned 3–8 nm nanoparticles and 40–45 nm edge length nanoprisms.

Table 1 lists also the surface-to-volume ratios S/V and average volumes $V_{1}$ calculated directly from the scattering intensities using Equations (2) and (3) (vide infra). When considering the S/V ratios, a sphere of 7.6 nm in diameter would have the same volume as determined for the particles in the initial solution. As the particles have the maximum diameter of about 10 nm, it means that at least part of the particles must be non-spherical or the system is polydisperse. An anisotropic 11 nm × 2 nm particle would also agree with the observed S/V and $V_{1}$. For the 150 min solution, we obtain only a slightly smaller S/V ratio than for the initial solution even though the average particle is interpreted to be significantly larger (35 to 45 nm in diameter). This indicates that the blue 150 min samples do not contain globular particles. Instead, the particles are flat or elongated compositions of the original particles of the initial solution. If the shape is triangular, then the thickness of the average triangle would be about 5 nm for a triangle with a side of 45 nm based on the volume in Table 1. Again, this picture agrees well with the cryo-TEM images.

**Figure 6:**
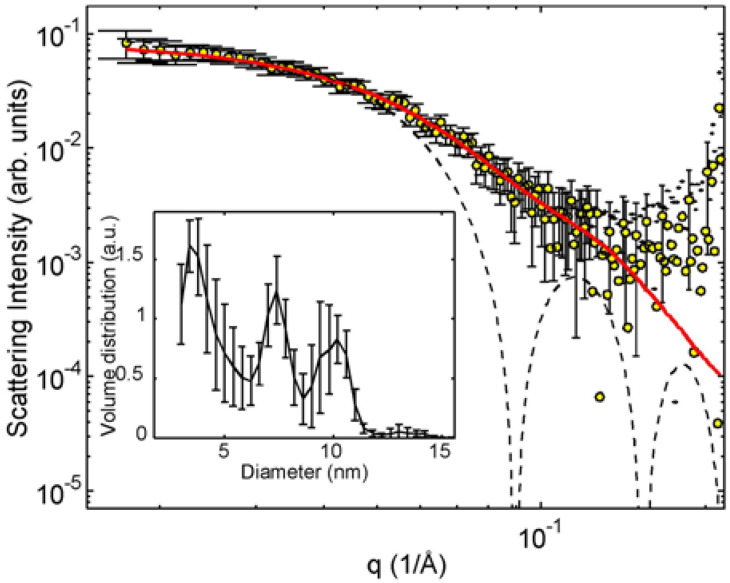
The scattering pattern of initial sample (yellow symbols) and theoretical scattering from a sphere with a diameter of 10 nm (dashed line). The inset shows the Monte Carlo (MC) simulated volume distribution of spheres in arbitrary units. It corresponds to the fit (full red line). See text for details.

Figure 6 shows the SAXS pattern of the initial sample and an alternative interpretation where the scattering curve stems from polydisperse spheres with pronounced average diameters around 7.4 nm and 10 nm. The volume distribution was defined as the number of particles of a certain size multiplied by their volume and obtained using an MC fitting of spheres after subtracting away the power law at small angles. The inset shows the central peak of the MC simulated particle distribution. This should be compared to the average particle volume that is calculated directly from the intensity (Table 1).

**Figure 7:**
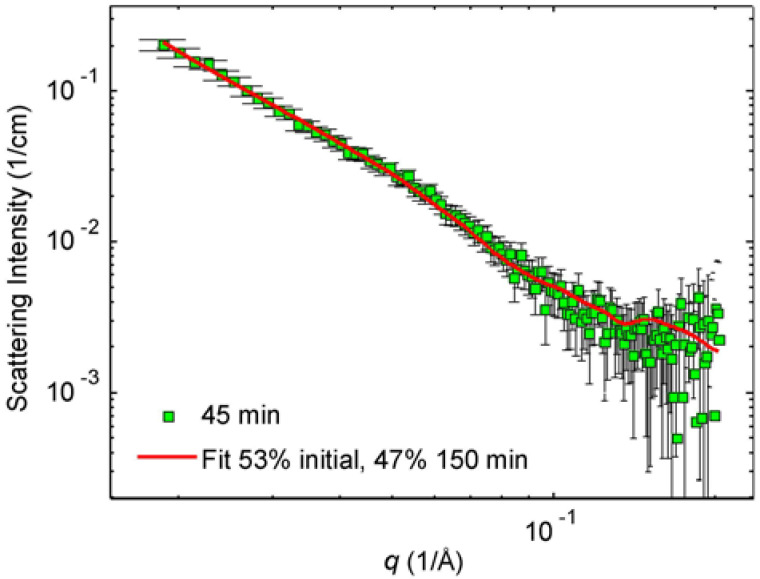
The scattering pattern of the 45 min sample (green squares). The solid red line is the corresponding “bimodal” model fit with the contribution of 53% of initial and 47% initial sample. See text for details.

The interpretation of the SAXS data of the 45 min irradiated sample is more difficult because of significant polydispersity assumed by cryo-TEM images. However, from the phenomenology of electron microscopy, we know that this sample is built on the compounds of the initial and 150 min sample. Similarly, the above-mentioned SAXS data of the initial and 150 min irradiated samples are understood to mirror nearly spherical initial particles and the nanoprisms with 40 nm edge length, respectively. We built thus a model where the intensity of the 45 min irradiated sample was fitted to the sum of the fits to the initial and the 150 min intensities by letting the proportions vary. By doing so, we found that the best fit corresponds to 53% scattering contribution from the initial sample and 47% contribution from the 150 min sample. Figure 7 shows the so-obtained fit to the SAXS pattern of the 45 min irradiated sample.

The percentage values given in Figure 7 should not be over-interpreted. In principle, if we know how many particles in the initial solution form one particle in the 150 min solution, we can estimate the relative number of each particle type in the solutions and then calculate the proportions of those particles in the 45 min solution. The volume of an average particle in the 150 min solution is 4600 nm^3^ and the volume of a (separated) average particle in the initial solution is 210 nm^3^. Therefore, the average particle in the 150 min blue solution consists of 4600/210~22 “initial” average particles. Considering the differences between indirectly determined values and the directly determined values, the relative error is about 20% corresponding to the deviation of ±4 particles. According to this reasoning, there are 22 times more particles in the initial solution compared to the final 150 min irradiated solution. So, if the 45 min sample is understood as a “mixture” of 53% of the initial solution and 47% of the 150 min solution, then about 22 of 23 particles would represent the initial solution and 1 of 23 final, the 150 min irradiated sample. This reasoning would also mean that the final particles arising from 150 min irradiation contain approximately two or three layers of initial particles.

## 3. Materials and Methods

Silver nanoparticles were prepared from AgNO_3_, sodium citrate, NaBH_4_ and bis(*p*-sulfonatophenyl)phenylphosphine) (BSPP) as described elsewhere [9]. For the photoinduced silver nanoprism growth, the aqueous silver nanoparticles solution was illuminated with 550 nm light up to 150 min. Three representative samples were studied: A sample before illumination and after 45 min and final 150 min illumination.

Specimens for electron microscopy were prepared in a controlled environment vitrification system (CEVS) to ensure stable temperature and to avoid loss of solution during sample handling. The specimens were prepared as thin liquid films, <300 nm thick, on lacey carbon filmed copper grids and plunged into liquid ethane at −180 °C. This led to vitrified specimens, avoiding component segmentation and rearrangement, and water crystallization. This means that the process preserves the original microstructures. The vitrified specimens were stored under liquid nitrogen until measured. An Oxford CT3500 cryoholder (High Wycombe, UK) and its workstation were used to transfer the specimen into the electron microscope (Philips CM120 BioTWIN Cryo, Eindhoven, the Netherlands) equipped with a post-column energy filter (Gatan GIF100, Pleasanton, CA, USA). The acceleration voltage was 120 kV. The images were recorded with a CCD camera under low electron dose conditions.

SAXS measurements were performed at the former beamline B1 of DORIS III synchrotron storage ring at DESY (Hamburg, Germany) [18]. The X-ray energies of 12 and 25 keV and the sample-to-detector distance of 3635 mm were used, leading to a *q* range of 0.006–0.2 Å^−1^. The beam size was 1 mm × 1 mm. The samples were measured in wax-sealed Hilgenberg glass mark tubes of about 4 mm inner diameter and 50 µm wall thickness. Hence the probed volume was about 4 mm^3^. The scattering patterns were measured using a 2-dimensional Gabriel-type multiwire proportional counter. The absolute intensity scale was calibrated using glassy carbon. The analysis followed the standard approach. The scattering intensity at zero angle I(0) is proportional to the number of scatterers N, to the square of the volume of one scatterer $V_{1}$ and to the square of the scattering length density difference Δρ between the scatterer and the surroundings as

(1) $$I(0)=N{\left(\Delta \rho \right)}^{2}V_{1}^{2}$$

In principle, information on the number of scatterers or the volume of scatterers can be obtained from I(0), if the other parameters are known. Here, the volume of one particle, $V_{1}$, was determined from I(0) and the invariant Q which is calculated as an integral

(2) $$Q=\int_{0}^{\infty }dqq^{2}I(q)=2\pi^{2}{\left(\Delta \rho \right)}^{2}V_{1}N$$

The surface-to-volume ratio S/V was calculated as

(3) $$\frac{S}{V}=\underset{q\to \infty }{lim}\frac{q^{4}I(q)}{Q}$$

The weighted autocorrelation function of the electron density of a particle is the distance distribution function, p(r), which is a measure of the particle shape. This function was calculated by indirect method using the GNOM software 2.7. (Hamburg, Germany) [19,20]. In this procedure, several maximum particle diameters were tried as an input parameter and the ones giving the best fits were chosen. This calculation was complemented by direct Fourier transform. In addition, the results for the 150 min illuminated sample expected to be prismatic were compared to the p(r) function calculated from a presupposed model of rigid triangular platelets. Particle polydispersity was further characterized by the Monte Carlo (MC) analysis of the size distribution as described in Ref. [21].

## 4. Conclusions

Where photoinduced growth of silver nanoparticles in water is concerned, the initial silver particles are interpreted as spheres with the radius of gyration of 3.4–3.6 nm, whereas the final nanoprisms are understood as triangles with the radius of gyration of 11–13 nm. The scattering data of the 45 min illuminated sample fit well to the model where the mixture contains roughly the same amount of initial nanoparticles and final nanoprisms. Though qualitative, these data combine a single-particle viewpoint and a macroscopic sample viewpoint on the nanoprism growth.

Future studies might include the use of grazing-incidence small-angle X-ray scattering as already demonstrated for nanoprisms prepared using sputtering [22]. They should also include the solid-state studies and, when micro- and nano-focussed X-ray beam are available, the micrometer size details in patterned structures such as microwires of silver composites [23]. Studies can also be extended to the particle aggregation when used in chemosensors [5]. One should also simulate nanoprism growth from the first principles and compare so obtained results to the scattering data.

## Figures and Tables

**Table 1:** Structural parameters of the initial solution and the 150 min illuminated solution estimated from small-angle X-ray scattering (SAXS) data by indirect GNOM and Fourier transform (FT) methods. *R*_g_ is the radius of gyration, *R*_max_ the maximum dimension, *S*/*V* the surface-to-volume ratio, and *V*_1_ the average volume of a single particle.

	Method					
	GNOM		FT			
Sample	*R*_g_ (nm)	*R*_max_ (nm)	*R*_g_ (nm)	*R*_max_ (nm)	*S*/*V* (m^−1^)	*V*_1_ (nm^3^)
0 min	3.63 ± 0.07	10	3.37 ± 0.15	11	1.36 × 10^9^	210
150 min	11.4 ± 0.5	34	13 ± 2	44	1.03 × 10^9^	4600
